# Targeted proteomics of extreme vascular phenotypes in type 1 diabetes: the ESCAPER study

**DOI:** 10.1186/s12933-026-03261-6

**Published:** 2026-06-20

**Authors:** Ola Ekström, Cecilia Kennbäck, Valeriya Lyssenko, Magnus Löndahl, Anders Christensson, Peter M. Nilsson, Anders Gottsäter, the ESCAPER Study Group

**Affiliations:** 1 Department of Clinical Sciences Malmö, Lund University, Lund, Sweden; 2 Department of Internal Medicine, Skåne University Hospital, Malmö, Sweden; 3 Department of Clinical Science, Mohn Center for Diabetes Research, University of Bergen, Bergen, Norway; 4 Department of Nephrology, Skåne University Hospital, Malmö, Sweden; 5 Department of Clinical Sciences Lund, Lund University, Lund, Sweden; 6 Eli Lilly Sweden AB, Helsingborg, Sweden

**Keywords:** Type 1 diabetes, Cardiovascular disease, Proteomics, Extreme phenotype, Vascular resilience, Biomarkers, von Willebrand factor, Endothelial function

## Abstract

Cardiovascular disease (CVD) is the leading cause of morbidity and mortality in Type 1 Diabetes (T1D), but a subset of individuals remains free from macrovascular or renal complications despite decades of hyperglycaemia and a significant risk factor burden. We used a targeted proteomic approach (Olink Cardiovascular panel III, targeting 92 proteins) to characterize the proteomic profile of cardiovascular resilience in T1D by comparing 92 patients with long-standing T1D (age 59.8 [53.2, 69.1], duration 40.0 [35.0, 45.2] years) free from macrovascular complications or nephropathy against a reference group of 57 T1D patients with accelerated vascular pathology (age 42.0 [32.0, 56.0], duration 22.0 [18.0, 27.0] years), proliferative retinopathy and/or nephropathy in relation to diabetes duration, termed Rapid Progressors (RP). Twenty proteins differed significantly between RP and Escapers (False Discovery Rate [FDR] < 0.05) after adjustment for age, sex, HbA1c, and eGFR: Caspase-3 was significantly higher in RP (Adjusted difference: + 2.12 Normalized Protein eXpression [NPX], *p* < 0.001). Proteins associated with platelet activation and leukocyte adhesion with increased levels in RP included Junctional Adhesion Molecule A (+ 1.40 NPX), Glycoprotein VI (GP6: + 1.29 NPX), and P-Selectin (+ 0.82 NPX) (all *p* < 0.001). PECAM-1 (+ 0.55 NPX) and TNFRSF14 (+ 0.43 NPX), were also elevated. RP also showed higher levels of metabolic and tissue-remodelling proteins; Transferrin Receptor (+ 0.53 NPX) and Fatty Acid Binding Protein 4 (+ 0.52 NPX), as well as higher Bleomycin Hydrolase, Trefoil Factor 3, GDF-15, U-PAR, and Cystatin B. Conversely, von Willebrand Factor (vWF) levels (− 1.35 NPX, *p* < 0.001) and Paraoxonase 3 (PON3) was lower in RP (− 0.34 NPX, *p* = 0.003). In conclusion, escaping complications in long-term T1D appears to be associated with active molecular mechanisms. Progression is marked by apoptosis (Caspase-3), fibrosis (CHI3L1) and platelet activation (GP6), whereas resilience is associated with a distinct signature involving higher vWF and PON3. These findings highlight a profound biological divergence between extreme T1D phenotypes and provide a foundation for further research into vascular resilience.

**Supplementary Information:**

The online version contains supplementary material available at 10.1186/s12933-026-03261-6.

**What is currently known?**

- CVD is the leading cause of mortality in type 1 diabetes (T1D).
- Some patients, termed ‘Escapers’, remain complication-free for decades.
- Traditional risk factors do not fully explain this vascular resilience.

**Key research question**

What proteomic signatures characterize cardiovascular resilience in T1D Escapers?

**What is new?**

- Discovery of a unique proteomic signature associated with the Escaper phenotype.
- A proteomic signature involving PON3 and vWF characterizes the Escaper phenotype, contrasting with the thrombo-inflammatory profile of rapid progressors.

**Clinical influence**

Defining the Escaper phenotype provides a framework for future protection research.

## Introduction

Despite significant advancements in glycaemic control and cardiovascular risk management, cardiovascular disease (CVD) remains the leading cause of morbidity and mortality in type 1 diabetes (T1D) [1]. While intensive metabolic control reduces this risk, a substantial residual risk persists, implicating molecular determinants not fully captured by glycaemic metrics alone [2–4]. Conversely, clinical experience and registry data identify a subset of individuals who, despite decades of hyperglycaemia and significant risk factor burden, remain free from macrovascular or renal complications.

These individuals, labelled as “Escapers”, challenge the traditional paradigm of diabetic complications driven solely by cumulative risk factor exposure [5]. We recently established the rationale for the ESCAPER study, hypothesizing that the absence of complications in these individuals is not merely due to the lack of risk factors, but potentially due to the presence of active protective mechanisms or “cardiovascular resilience” [6]. While traditional risk markers/factors such as HbA1c and lipids explain a large proportion of variance, they fail to fully capture the biological heterogeneity seen in long-standing T1D survivors [7]. 

Pioneering initiatives, most notably the Joslin Medalist Study [4, 8, 9], followed by Scandinavian efforts such as the PROLONG and DIALONG studies [10–12], and the FinnDiane 50-year cohort [13], have begun to characterize these survivors, suggesting roles for specific genetic and metabolic factors. However, the comprehensive molecular architecture underlying this resilience, particularly regarding circulating proteomic signatures, remains largely unexplored. To date, most biomarkers identified in T1D are markers of damage (e.g., endothelial dysfunction, inflammation) identified in cohorts with established disease [14]. Few studies have utilized an “extreme phenotype” approach to isolate signals of protection by contrasting those who remain complication-free despite long diabetes duration against those with an accelerated disease trajectory (“Rapid Progressors”).

In this exploratory analysis of the ESCAPER cohort [6], we utilized a targeted proteomic approach (Olink Cardiovascular panel III) to characterize the proteomic profile of cardiovascular resilience. By comparing T1D patients with > 30 years of complication-free duration to a phenotype of accelerated vascular pathology, so called Rapid progressors (RP), we aimed to identify circulating proteins that differentiate vascular stability from vascular injury. Specifically, we sought to determine if T1D Escapers exhibit a distinct proteomic profile, providing a foundation for future studies investigating endogenous vascular resilience.

## Methods

### Study design and participants

This cross-sectional study applied an “extreme phenotype” design to identify biomarkers of vascular protection. The study population included two T1D cohorts representing opposing disease trajectories:

- The ESCAPER Cohort (*n* = 113): As described previously [6], this group comprised individuals with long-standing T1D (> 30 years) free from macrovascular complications (coronary heart disease, cerebrovascular disease, aortic dissection, peripheral arterial disease), proliferative retinopathy and diabetic nephropathy. Background retinopathy or microalbuminuria were not exclusion criteria. After excluding individuals with missing baseline covariates, the final analytical sample comprised 92 individuals.
- The PROLONG Cohort (*n* = 58): A reference group of “Rapid Progressors” (RP) was derived from the PROLONG study [11] specifically selecting the sub-cohort characterized by accelerated complication progression (presenting with proliferative retinopathy and/or nephropathy relative to diabetes duration). After excluding individuals with missing baseline covariates, the final analytical sample comprised 57 individuals.

### Clinical and biochemical assessments

 Detailed clinical phenotyping was performed at Skåne University Hospital (Escapers) and participating centres (PROLONG) according to standardized protocols. Routine biochemical markers, including HbA1c and lipid profiles, were analysed at certified clinical chemistry laboratories using standard accredited methods.

### Proteomic analysis

 Proteomic profiling was performed using the Olink^®^ Target 96 Cardiovascular III panel (Olink Proteomics, Uppsala, Sweden). This panel targets 92 proteins integral to cardiovascular biology, inflammation, and metabolism. The analysis utilizes Proximity Extension Assay (PEA) technology, which combines the specificity of antibody-based detection with the sensitivity of PCR. Protein expression levels are reported as Normalized Protein Expression (NPX) values on a log2 scale, where a 1-unit increase represents a doubling of protein concentration. Quality control (QC) was performed according to the manufacturer’s standards. Proteins were evaluated based on detectability and assay precision.

###  Statistical analysis

 Baseline characteristics were compared using the Wilcoxon rank-sum test for continuous variables and Chi-square tests for categorical variables. Protein expression (NPX) was compared between groups using multivariable linear regression (ANCOVA), adjusting for age, sex, HbA1c, and eGFR. Age was prioritized as a covariate over diabetes duration for cumulative exposure to avoid multicollinearity, and eGFR was included as renal clearance confounds endogenous protein levels [15].

To control for the false discovery rate (FDR) inherent in high-throughput proteomics, *p*-values from the adjusted models were corrected using the Benjamini-Hochberg method. An FDR (q-value) < 0.05 was considered statistically significant.

All statistical analyses were performed using R version 4.5.2. Multivariable linear models (ANCOVA) and Benjamini-Hochberg false discovery rate (FDR) corrections were computed using the base R stats package.

### Ethics

 All investigations in the project have been ethically approved by The Swedish Ethical Review Authority (Dnr. 2021 − 01809, 2022-00968-02, and 2023-05419-02) for the ESCAPER study, and the Regional Ethics Review Board, Lund, Sweden (Dnr 777/2009) for the PROLONG study. Informed consent is a prerequisite for participation in the study and includes consent for publication.

## Results

## Baseline characteristics

 Clinical characteristics of the analytical sample are presented in Table 1. The Escaper cohort (*n* = 92) had a median diabetes duration of 40.0 [IQR 35.0, 45.2] years, compared to 22.0 [IQR 18.0, 27.0] years in the RP cohort (*n* = 57). Escapers were significantly older (*p* < 0.001), and had lower HbA1c (*p* < 0.001) and eGFR levels (*p* < 0.001) compared to RP. There were no significant differences in sex distribution between the groups.

**Table 1:** Clinical characteristics of the analytical sample

Variable	Escapers (*n* = 92)	Rapid progressors (*n* = 57)	*P*-value
Age (years)	59.8 [53.2, 69.1]	42.0 [32.0, 56.0]	< 0.001
Sex			0.270
Male	55 (59.8%)	28 (49.1%)	
Female	37 (40.2%)	29 (50.9%)	
Diabetes duration (years)	40.0 [35.0, 45.2]	22.0 [18.0, 27.0]	< 0.001
HbA1c (mmol/mol)	51.0 [46.0, 57.2]	75.0 [64.0, 87.0]	< 0.001
eGFR (mL/min/1.73 m^2^)	71.5 [62.0, 86.0]	97.2 [78.6, 116.5]	< 0.001
BMI (kg/m^2^)	25.2 [23.0, 27.8]	25.5 [22.8, 28.1]	0.807
Systolic blood pressure (mmHg)	131.0 [122.0, 139.2]	126.2 [115.0, 138.1]	0.087
Diastolic blood pressure (mmHg)	74.0 [69.0, 78.0]	78.5 [70.5, 82.8]	0.004
LDL cholesterol (mmol/L)	2.3 [1.9, 2.7]	2.7 [2.3, 3.4]	< 0.001
Triglycerides (mmol/L)	0.7 [0.6, 0.9]	0.9 [0.7, 1.2]	< 0.001

Values are presented as Median [IQR] for continuous variables and n (%) for categorical variables.* P*-values are calculated using Wilcoxon rank-sum test for continuous variables and Chi-square test for categorical variables

## Proteomic analysis

 All 92 proteins included in the Olink Cardiovascular III panel passed quality control measures and were detected above the Limit of Detection (LOD) in > 75% of samples, with high analytical precision (mean intra-assay CV 6%).

To isolate the association with the “Escaper” phenotype, a multivariable linear regression model adjusted for age, sex, HbA1c, and eGFR, was applied.

**Fig. 1:**
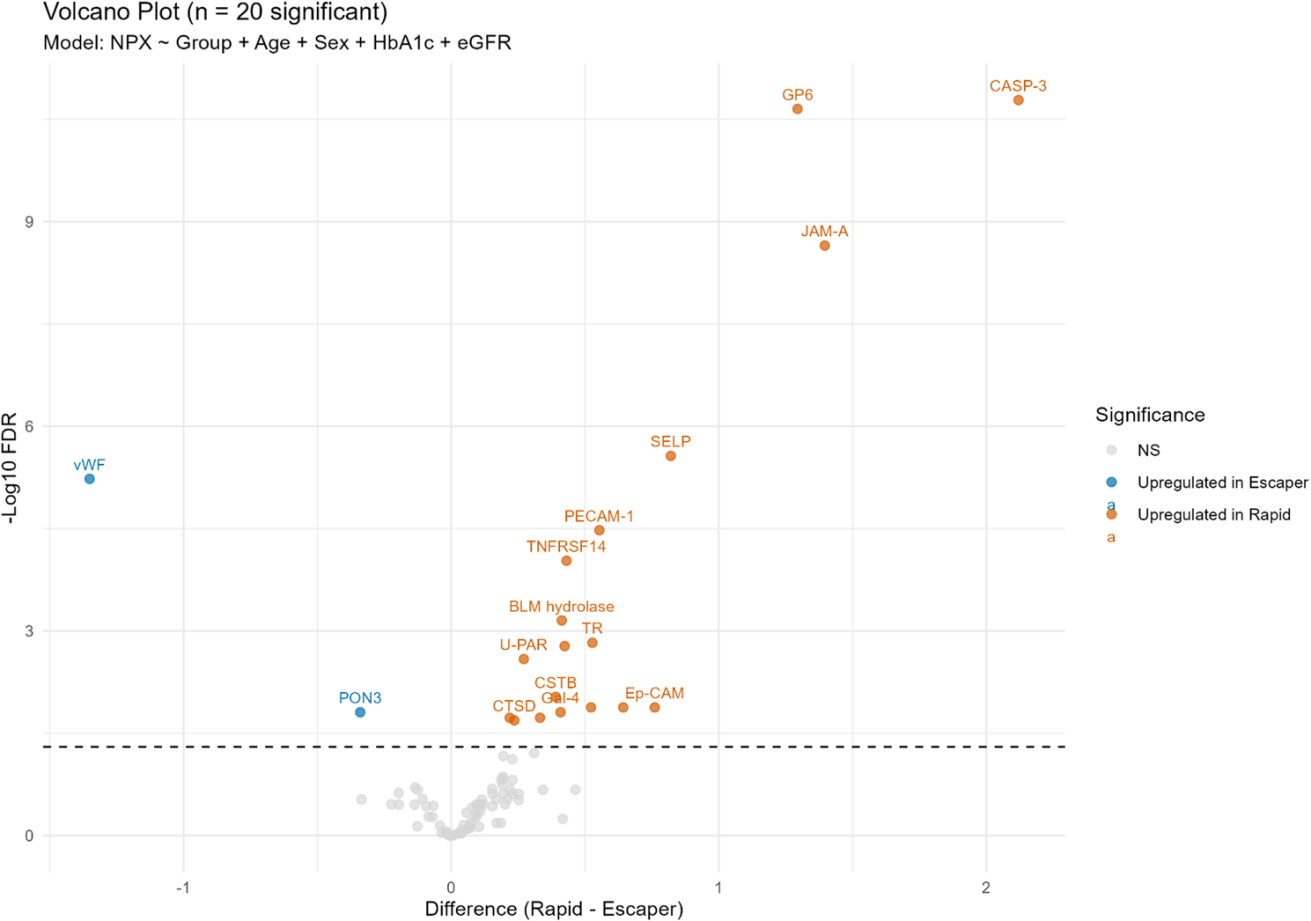
Volcano plot of adjusted protein expression differences. The x-axis displays the log2 fold change (coefficient from the linear model), where positive values = higher levels in rapid progressors (RP), and negative values = higher levels in Escapers. The y-axis represents the -Log10 adjusted *p*-value (FDR). Proteins coloured in orange show significantly higher levels in RP (e.g., CASP-3, JAM-A), while proteins in blue show higher levels in Escapers (vWF, PON3), adjusted for age, sex, eGFR, and HbA1c

The global profile of differentially regulated proteins is visualized in Fig. 1. In total, 20 proteins were significantly differentially expressed between RP and Escapers (FDR < 0.05) in the fully adjusted model (Table 2).

**Table 2:** Differentially regulated proteins in rapid progressors (RP) versus escapers

Protein	Adjusted difference (95% CI)	*P*-value	FDR (q-value)	Association
Caspase-3 (CASP-3)	+ 2.12 (1.61 to 2.64)	< 0.001	< 0.001	Higher in RP
Glycoprotein VI (GP6)	+ 1.29 (0.97 to 1.61)	< 0.001	< 0.001	Higher in RP
JAM-A	+ 1.40 (1.01 to 1.79)	< 0.001	< 0.001	Higher in RP
P-Selectin (SELP)	+ 0.82 (0.53 to 1.11)	< 0.001	< 0.001	Higher in RP
von Willebrand Factor (vWF)	− 1.35 (− 1.86 to − 0.86)	< 0.001	< 0.001	Higher in Escapers
PECAM-1	+ 0.55 (0.33 to 0.77)	< 0.001	< 0.001	Higher in RP
TNFRSF14	+ 0.43 (0.25 to 0.62)	< 0.001	< 0.001	Higher in RP
BLM Hydrolase	+ 0.41 (0.22 to 0.61)	< 0.001	0.001	Higher in RP
Transferrin receptor (TR)	+ 0.53 (0.27 to 0.80)	< 0.001	0.001	Higher in RP
Trefoil factor 3 (TFF3)	+ 0.42 (0.21 to 0.65)	< 0.001	0.002	Higher in RP
U-PAR	+ 0.27 (0.13 to 0.42)	< 0.001	0.003	Higher in RP
Cystatin B (CSTB)	+ 0.39 (0.16 to 0.63)	0.001	0.009	Higher in RP
FABP4	+ 0.52 (0.19 to 0.86)	0.002	0.013	Higher in RP
Ep-CAM	+ 0.76 (0.27 to 1.23)	0.002	0.013	Higher in RP
Galectin-4 (Gal-4)	+ 0.41 (0.15 to 0.68)	0.003	0.016	Higher in RP
CHI3L1 (YKL-40)	+ 0.64 (0.23 to 1.05)	0.002	0.013	Higher in RP
Paraoxonase 3 (PON3)	− 0.34 (− 0.56 to − 0.11)	0.003	0.016	Higher in Escapers
GDF-15	+ 0.33 (0.11 to 0.56)	0.004	0.019	Higher in RP
Chemerin (RARRES2)	+ 0.22 (0.07 to 0.37)	0.004	0.019	Higher in RP
Cathepsin D (CTSD)	+ 0.24 (0.07 to 0.40)	0.004	0.020	Higher in RP

Only proteins with a false discovery rate (FDR) < 0.05 are shown. Adjusted difference: difference in normalized protein expression (NPX) representing rapid progressors (RP) relative to escapers, adjusted for age, sex, HbA1c, and eGFR. Positive values indicate higher levels in RP; negative values indicate higher levels in Escapers

**Fig. 2:**
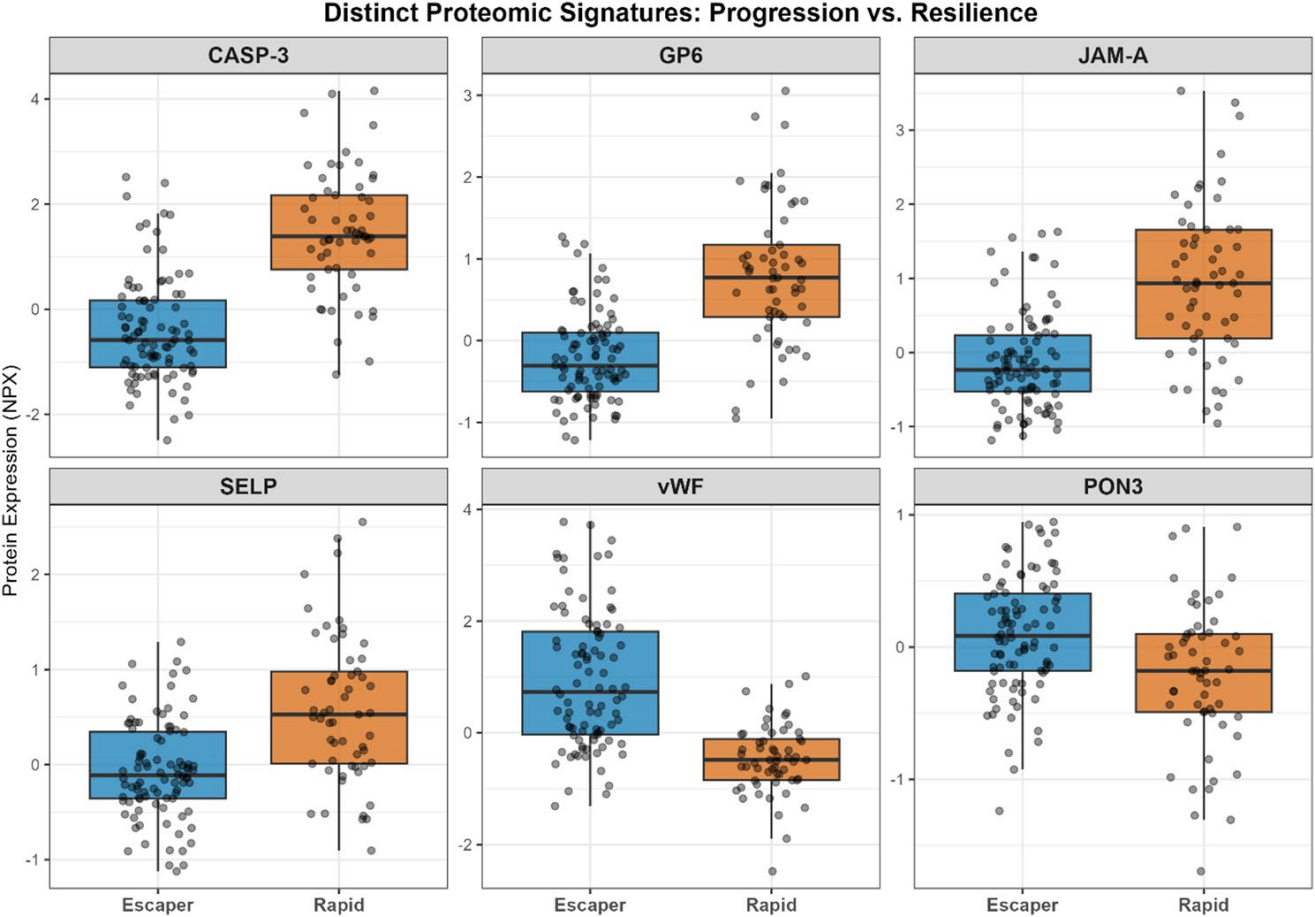
Distribution of key differentially regulated proteins. Boxplots displaying the normalized protein expression (NPX) levels for six selected proteins in Escapers (blue) versus rapid progressors, RP (orange). The central line represents the median, box limits indicate the interquartile range (IQR), and whiskers extend to 1.5x IQR. Individual data points are overlaid to visualize distribution. CASP-3, Caspase-3; GP6, Glycoprotein VI; JAM-A, Junctional adhesion molecule A; PON3, Paraoxonase 3; SELP, P-Selectin; vWF, von willebrand factor

Levels of Caspase-3 (CASP-3) were significantly higher in RP compared to Escapers (Adjusted difference: + 2.12 NPX, *p* < 0.001). The distribution of NPX values for Caspase-3 and other key markers is presented in Fig. 2. Similarly, proteins associated with platelet activation and leukocyte adhesion were markedly higher in Rapid Progressors, including Junctional Adhesion Molecule A (JAM-A: + 1.40 NPX), Glycoprotein VI (GP6: + 1.29 NPX), and P-Selectin (SELP: + 0.82 NPX) (all *p* < 0.001). Other inflammatory markers, including PECAM-1 (+ 0.55 NPX) and TNFRSF14 (+ 0.43 NPX), were also elevated.

Beyond inflammatory pathways, elevated levels of metabolic and tissue-remodelling proteins were observed in RP, including Transferrin Receptor (TR: + 0.53 NPX) and Fatty Acid Binding Protein 4 (FABP4: + 0.52 NPX). Additional proteins showing higher levels included Bleomycin Hydrolase, Trefoil Factor 3 (TFF3), GDF-15, U-PAR, and Cystatin B (CSTB).

Conversely, levels of von Willebrand Factor (vWF) were significantly lower in RP compared to Escapers (Adjusted difference: − 1.35 NPX, *p* < 0.001). Paraoxonase 3 (PON3) was the only other protein sharing this inverse association, being higher in Escapers (Adjusted difference: − 0.34 NPX, *p* = 0.003).

Proteins that showed significant differences in unadjusted analyses, but lost significance after adjustment for eGFR and age, include Tissue Plasminogen Activator (t-PA), Notch 3, and IGFBP-2 (*p* > 0.05). Unadjusted univariate comparisons for all 92 proteins are provided in Supplementary Table 1.

## Discussion

In this exploratory study using targeted proteomics, we identified a molecular signature characterizing “Escapers”, individuals with long-standing T1D who possess resilience to macrovascular diabetes complications and nephropathy. By contrasting these survivors against the RP phenotype, we identified two opposing molecular profiles: a canonical injury signature apoptosis and platelet activation in progressors, versus a specific profile involving von Willebrand Factor (vWF) and Paraoxonase 3 (PON3) in Escapers. This resilience profile aligns conceptually with findings from the Joslin Medalist cohort, where vascular protection was linked to specific antioxidative signatures, including the PON3 homolog PON1 [9].

The proteomic profile of RP provided a robust biological validation of our “high-risk” cohort definition. The most significantly differentially abundant protein, Caspase-3, confirms active apoptotic cell death. This finding aligns with previous reports, where elevated circulating Caspase-3 was strongly associated with incident diabetes and coronary artery disease risk [16]. Beyond apoptosis, the RP phenotype was defined by markers of endothelial stress and thrombotic risk. We observed elevated levels of Glycoprotein VI (GP6) and P-Selectin (SELP), key mediators of platelet activation and leukocyte rolling [17, 18], alongside PECAM-1. This suggests a vasculature in a state of ongoing thrombo-inflammation, a mechanism recently highlighted by Galli et al. as a key driver of residual cardiovascular risk in coronary artery disease [2], yet evident decades before the typical onset of macrovascular events. Furthermore, markers associated with adverse remodelling were prominent. GDF-15, a prognostic marker for heart failure and renal decline [19, 20], was significantly elevated in RP, as were the fibrosis-associated protein CHI3L1 (YKL-40) and the urokinase receptor U-PAR, a strong predictor of progressive nephropathy [21, 22]. Metabolic stress was also evident through elevated FABP4 [23], linking adipocyte dysfunction to the vascular pathology observed in this group.

In contrast, the T1D Escaper cohort was characterized by higher levels of vWF and PON3. We recognize that elevated vWF is conventionally viewed as a marker of endothelial damage, chronic inflammation, and a predictor of future vascular events [24–27]. However, in the present study, this elevation among Escapers occurs without concurrent established injury and platelet activation markers (e.g., Caspase-3, SELP, GP6). In this specific, long-term complication-free cohort, elevated vWF likely reflects preserved adaptive endothelial function rather than injury [28]. Importantly, the Olink assay measures total vWF antigen, not activity or multimer profiles. Finally, as causality cannot be determined in this cross-sectional design, the exact implications of elevated vWF in relation to resilience require further longitudinal investigation. Beyond the complex vWF signal, the Escaper phenotype was also associated with elevated levels of PON3, an enzyme localized to HDL and mitochondria. PON3 exerts lactonase activity that prevents lipid oxidation and stabilizes mitochondrial function, thereby neutralizing oxidative stress [29, 30]. Together, the combined elevation of vWF and PON3 provides a distinct molecular signature characterizing the Escaper phenotype. Rather than proving a direct causal mechanism, this profile contrasts with the active injury signature observed in Rapid Progressors. This contrast suggests a coordinated ability to manage chronic metabolic stress without triggering thrombo-inflammation, reinforcing the validity and utility of our extreme phenotype model.

The primary strength of this study is the “extreme phenotype” design, allowing us to filter out biological noise and isolate resilience factors obscured in more general cohorts of so-called Medallists that include also survivors of previous events. The alignment of our Rapid Progressor findings with established literature validates the platform.

However, limitations exist. First, the targeted nature of the Olink panel biases detection towards known cardiovascular proteins. While the identification of vWF and PON3 within this disease-centric panel highlights their robust association with the Escaper phenotype, future studies utilizing untargeted proteomics (e.g., mass spectrometry) are warranted to capture the full breadth of the resilience proteome.

Second, the cross-sectional design precludes causal inferences; differentially abundant proteins may represent downstream consequences of existing tissue injury. However, capturing this in vivo landscape serves the study’s exploratory aim: to generate mechanistic hypotheses. Furthermore, while adjustment for age, sex, HbA1c, and eGFR helps mitigate baseline imbalances, it does not fully eliminate residual confounding. Escapers represent a highly selected population of long-term survivors. Survivorship bias is inherent to this extreme phenotype design and is necessary to enrich for resilience signals. Therefore, our findings apply to long-duration T1D patients with extreme phenotypes and may not extend to unselected populations. Consequently, these signatures require validation in an independent T1D cohort. Data availability also limits the analysis. A single-point HbA1c assessment restricts the evaluation of cumulative metabolic burden. Similarly, the influence of differential treatment exposure during a long duration of diabetes cannot be ruled out. Medication at the date of sampling does not always reflect long-term pharmacological exposure. Even with access to such data, the inherent bi-directional confounding, possibly driven by confounding by indication in progressors and healthy user bias in Escapers, would complicate the interpretation of cross-sectional associations. Whether medications explain the divergent vWF and PON3 levels therefore remains an important consideration for future investigations.

## Conclusion

Escaping major diabetes complications in long-term T1D is associated with a distinct molecular signature rather than simply the absence of injury markers. While progression is marked by apoptosis (Caspase-3), fibrosis (CHI3L1), and platelet activation (GP6), resilience is associated with a distinct circulating profile characterized by antioxidative defence (PON3) and altered endothelial activity (vWF). These findings highlight the distinct biological divergence between extreme T1D phenotypes and provide a foundation for future longitudinal studies investigating endogenous vascular resilience.

## Electronic Supplementary Material

Below is the link to the electronic supplementary material.

Supplementary Table 1

## Data Availability

The datasets generated during the current study are not publicly available due to restrictions under European data protection legislation (GDPR) and the Swedish Ethical Review Act regarding the sharing of sensitive personal health data. However, pseudonymized data are available from the corresponding author upon reasonable request, subject to a formal data sharing agreement and ethical approval.
